# Needle Arthroscopy for Osteochondral Lesions of the First Metatarsophalangeal Joint: A Standardized Approach

**DOI:** 10.1016/j.eats.2023.02.041

**Published:** 2023-06-05

**Authors:** Alex B. Walinga, Jari Dahmen, Tobias Stornebrink, Gino M.M.J. Kerkhoffs

**Affiliations:** a Amsterdam UMC location of the University of Amsterdam, Department of Orthopedic Surgery and Sports Medicine, Amsterdam, The Netherlands; b Amsterdam Movement Sciences, Musculoskeletal Health, Sport, Amsterdam, The Netherlands; c Academic Center for Evidence-Based Sports Medicine, Amsterdam, The Netherlands; d Amsterdam Collaboration for Health & Safety in Sports, International Olympic Committee Research Center, Amsterdam, The Netherlands

## Abstract

Cartilage and osteochondral lesions of the first metatarsophalangeal (MTP-1) joint are characterized by pain during weight bearing and walking. The lesions often require surgical intervention(s). Arthroscopic bone marrow stimulation may be considered the preferred operative intervention for small lesions. Technological advances, patient preferences, and economic considerations combine to foster the development of minimally invasive needle arthroscopic procedures. This technical note presents and highlights our minimally invasive surgical technique for needle arthroscopic treatment through bone marrow stimulation for osteochondral lesions of the MTP-1 joint.

## Introduction

Over the years, possibilities for metatarsophalangeal (MTP) joint arthroscopy have evolved from diagnostic inspection to interventional procedures.^1^ The advancement of small-joint arthroscopy has made it possible for orthopedic surgeons to treat intra-articular pathologies using a minimally invasive approach, while simultaneously reducing the risk of iatrogenic injury. MTP-1 arthroscopy is currently used for the treatment of hallux rigidus, (osteo)chondral lesions of the phalanx and/or the metatarsal head, nonunions, loose bodies, synovitis, (bacterial) arthritis, and drainage of gouty tophi. It can be stated that further innovation will arise from needle arthroscopy, which gained popularity as a feasible technique for accurately diagnosing and treating a wide range of intra-articular pathologies in a variety of joints and patients, including the MTP-1.^2^, ^3^, ^4^, ^5^ Indications and contraindications are listed in Table 1. The present technical note aims to describe a standardized approach to needle arthroscopy of the first MTP joint using the needle arthroscopy system (NanoScope; Arthrex, Naples, FL) for osteochondral lesions of the MTP-1 joint. The approach ensures and facilitates a safe, uniform, and beneficial adoption and adherence to the emergent technique.

**Table 1:** Indications and Contraindications

Indications	Contraindications
Symptoms for at least 6 months	Presence of big osteophytes preventing a proper visualization of the MTP-1 joint
Failure of an adequate conservative treatment protocol	No adequate conservative protocol followed yet
Deep pain in/around the MTP-1 joint during walking/weight bearing, which decreases in or during rest	Decreased or inadequate vascular status
	Soft-tissue compromise
	Relative contraindication: previous surgery or surgeries to the MTP-1 joint
	Severe joint-space narrowing

MTP, metatarsophalangeal.

The study was conducted in agreement with the 1964 Helsinki Declaration and its later amendments. Ethical approval by our institution’s review board was not required.

## Surgical technique

Video 1 presents the technique through a step-by-step approach. Real-life intraoperative videos are presented in the present article in order to display the technique.

### Equipment

The needle arthroscopy equipment (NanoScope; Arthrex) is divided into two parts: the sterile disposable handpiece set and the portable video console. A semirigid, zero-degree needle arthroscope, sharp and blunt obturators, and appropriate sheaths are included in this handpiece set.^2^^,^^3^

### Patient Setup

**Fig 1:**
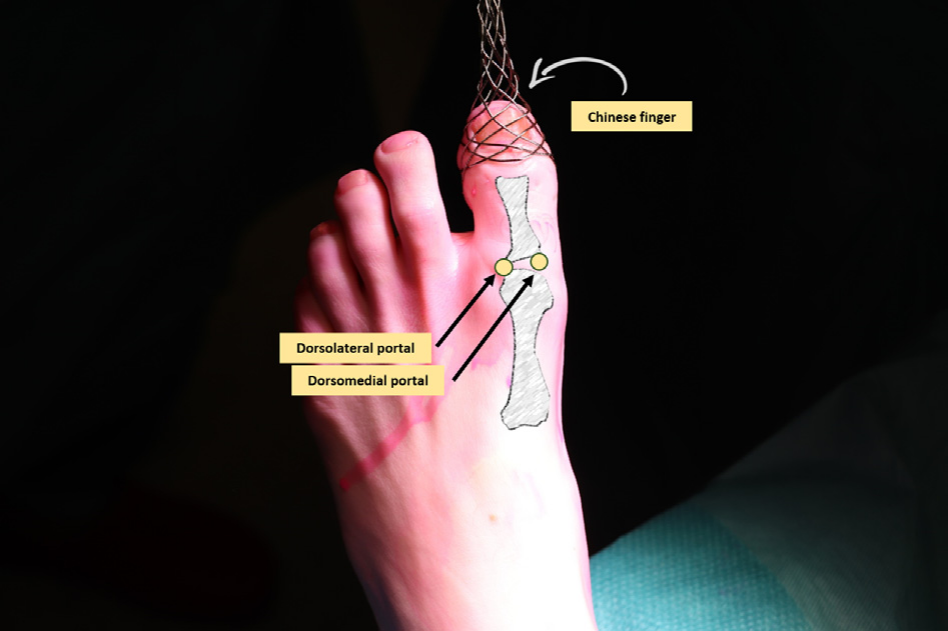
Left foot, seen from a dorsal perspective, with the first metatarsophalangeal joint in distraction using a Chinese finger and illustration of the proximal phalanx and metatarsal head. The dorsomedial and dorsolateral portals are marked for the needle arthroscopic approach.

The patient is positioned in a supine position on a standard operating table, with the foot at the edge of the bed. The surface anatomy of the foot is marked out, including the metatarsal head and the base of the proximal phalanx, as well as the portal locations (Fig 1). Depending on the planned operative procedure, a tourniquet may be applied at the thigh and is inflated to 250 mm Hg. The surgical field is disinfected with a chlorohexidine solution and covered with sterile draping. Subsequently, a Chinese finger with a distractor is applied for the distraction of the MTP-1 joint (Fig 1).

### Portal Placement, Arthroscope Introduction, and Joint Distention

**Fig 2:**
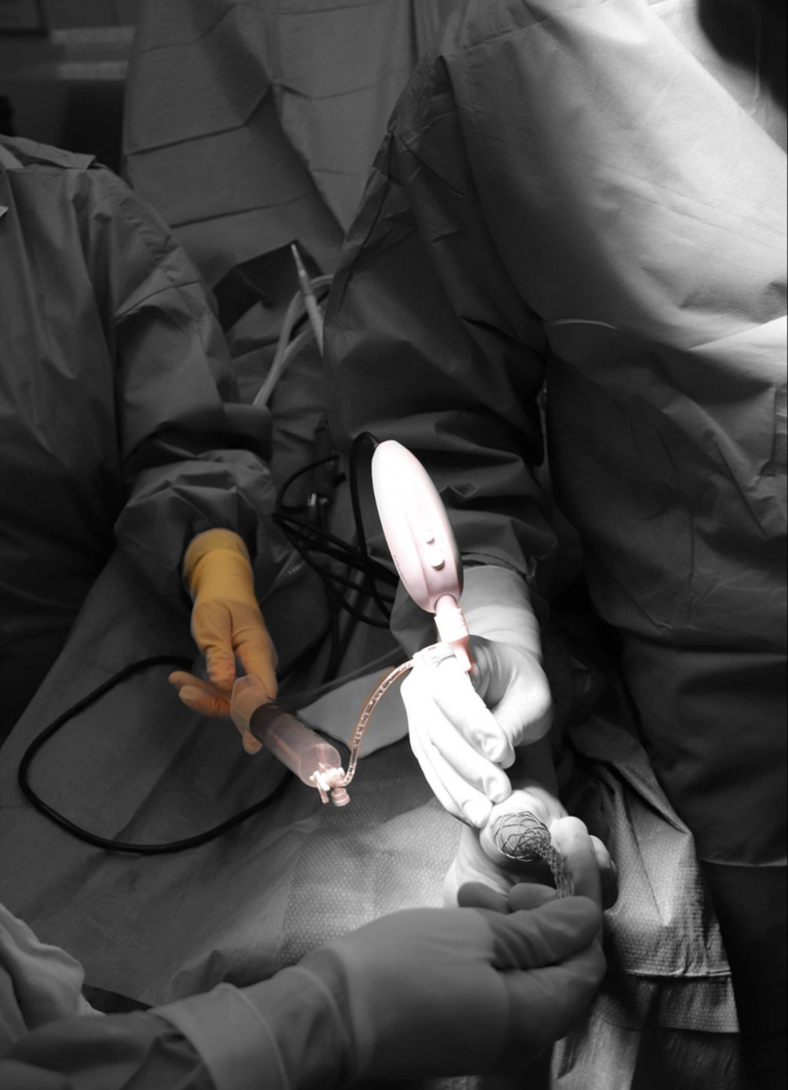
Left foot, seen from an anterior perspective. A 50-cc syringe, filled with sterile saline, is connected to a 3-way tap, which is then connected to the cannula. This will allow you to properly visualize the first metatarsophalangeal joint by distending the joint.

**Fig 3:**
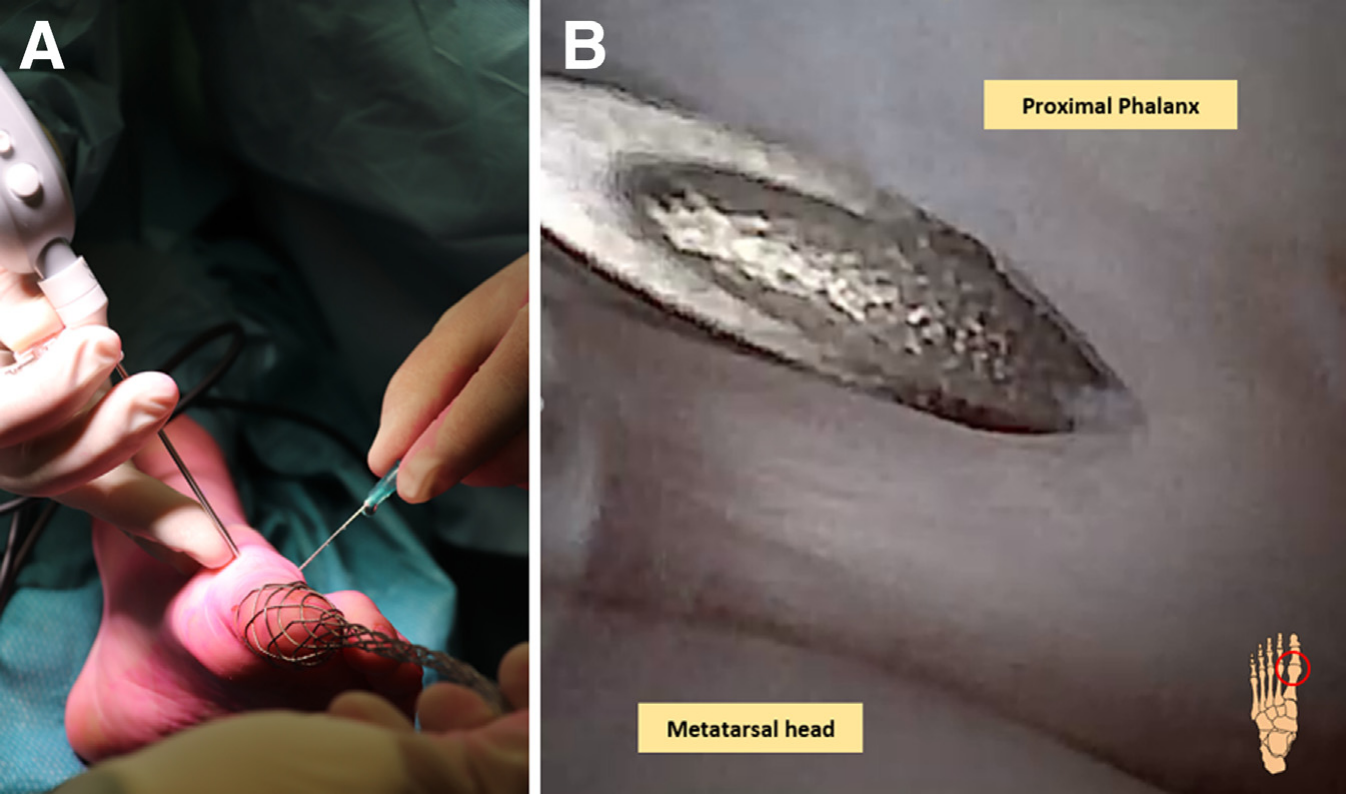
(A and B) Left foot, seen from an anterior, dorsal perspective, with the first metatarsophalangeal joint in distraction using a Chinese finger. The dorsolateral portal is first confirmed with a 21G (green needle) under visualization of the needle arthroscopy seen from the dorsomedial portal.

To ensure adequate visualization of the first MTP joint, portal placement is essential. The dorsomedial and lateral portals are located and marked. First, the dorsomedial portal is made. The skin is prepared with a 2-mm stab incision, a 2.2-mm diameter cannula is then loaded with a blunt obturator, and the cannula is penetrated through the joint capsule and entered intra-articularly. Slight noninvasive distraction may be helpful in achieving intra-articular positioning (Fig 1). The obturator is then removed from the cannula and replaced with the 1.9-mm diameter needle arthroscope. This needle arthroscope is semirigid and has a 0° direction of view.^2^^,^^3^ A 50-cc syringe is connected to a 3-way tap, which is connected to the cannula, and the joint can be distended with sterile saline (Fig 2). The dorsolateral portal can now be established under direct intra-articular visualization from the needle arthroscopy. Proper positioning is confirmed by the visual introduction of a 21G (green) needle (Fig 3, A and B). Further steps of the procedure concerning the dorsolateral portal are similar to those of the dorsomedial portal through the usage of a 2-mm stab incision of the skin and a cannula loaded with a blunt obturator to penetrate the joint capsule.

### Inspection

**Fig 4:**
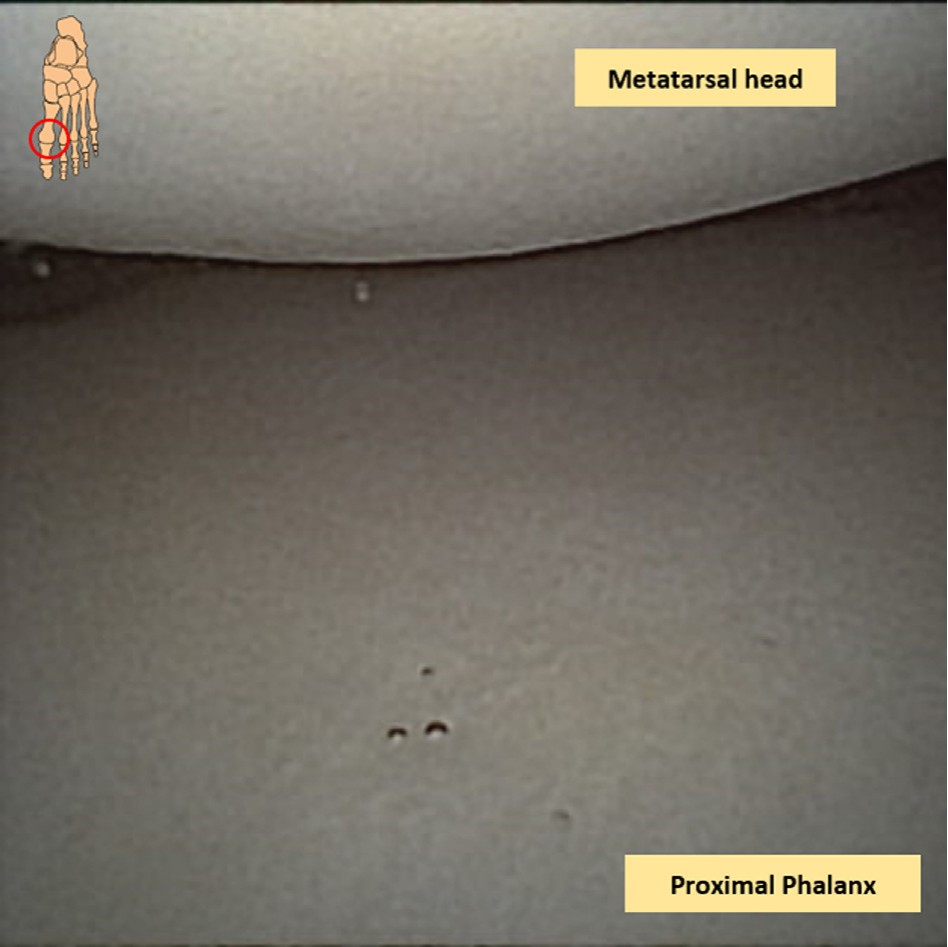
Needle arthroscopic intra-articular view of the first metatarsophalangeal joint with the metatarsal head and the proximal phalanx.

**Fig 5:**
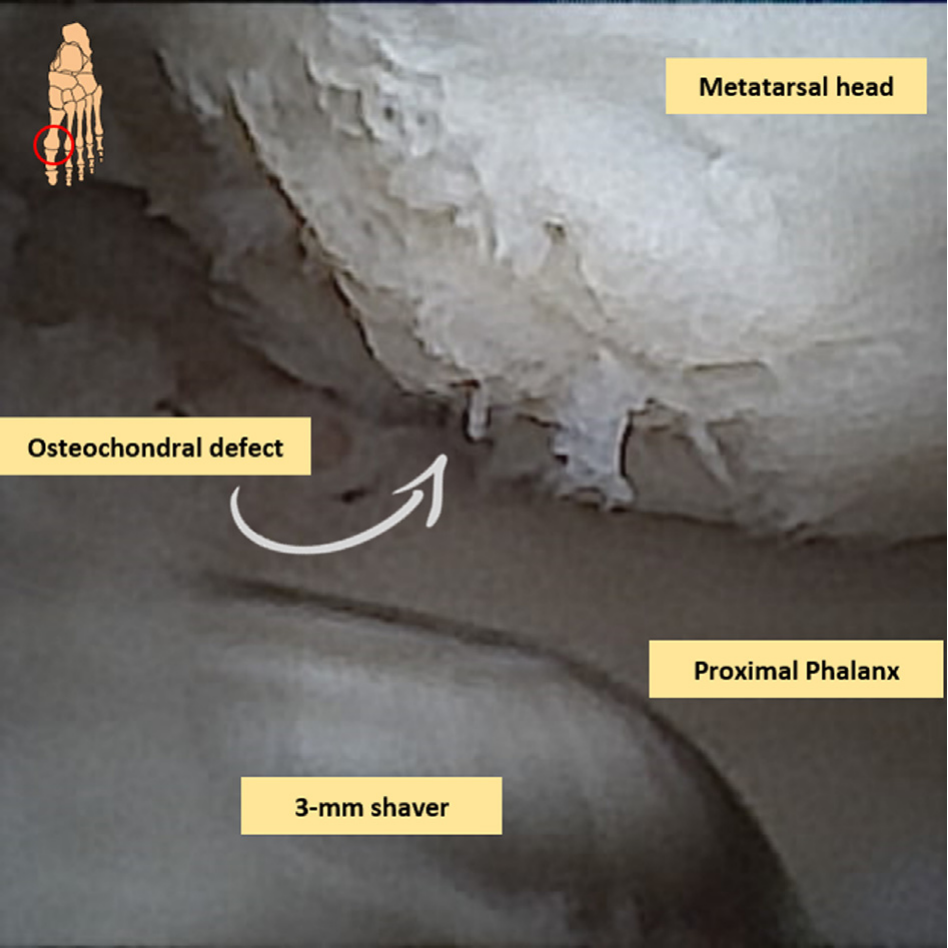
Intra-articular (osteo)chondral lesion of the metatarsal head seen with needle arthroscopy. A 3-mm shaver blade is inserted to debride the osteochondral defect until healthy borders of the surrounding cartilage are reached.

A thorough inspection of the MTP-1 joint can now be performed. Holding the arthroscope distally on its camera tube and with a pencil grip will aid in maintaining stability with this delicate equipment. The inspecting is initiated with the assessment of macroscopic evaluation of the metatarsal head, proximal phalanx, medial and lateral gutters, as well as the joint capsule (Fig 4; Video 1). Attention is paid to the presence of synovitis, osteophytes, and (osteo)chondral lesions. The present osteochondral defect—either on the phalangeal side of the joint or on the metatarsal side of the joint—is inspected with regard to the macroscopic appearance, quality of the cartilage, as well as size of the lesion (Fig 5). Once the examination has been completed, the intervention can be started.

### Intervention

**Fig 6:**
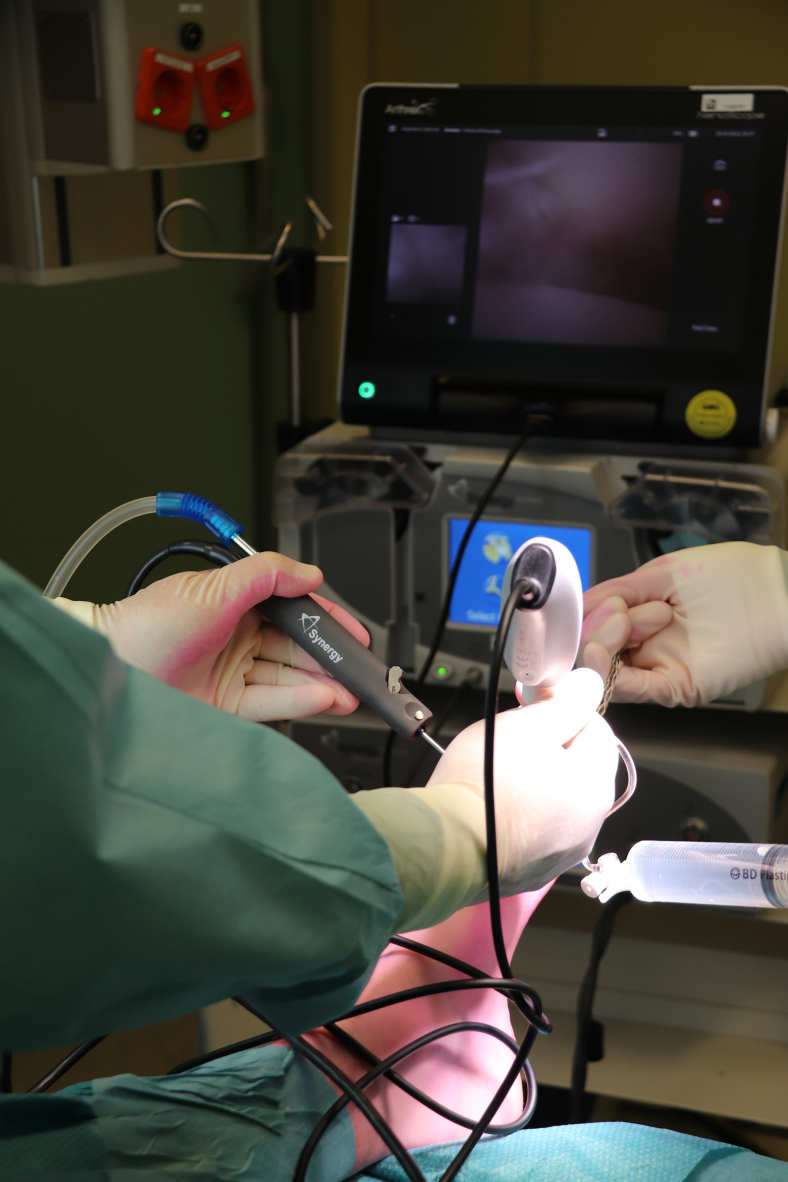
View from a dorsal perspective. Debridement and nettoyage of the first metatarsophalangeal joint can be performed with a small (2 or 3 mm) shaver blade under visualization of the needle arthroscopy.

**Fig 7:**
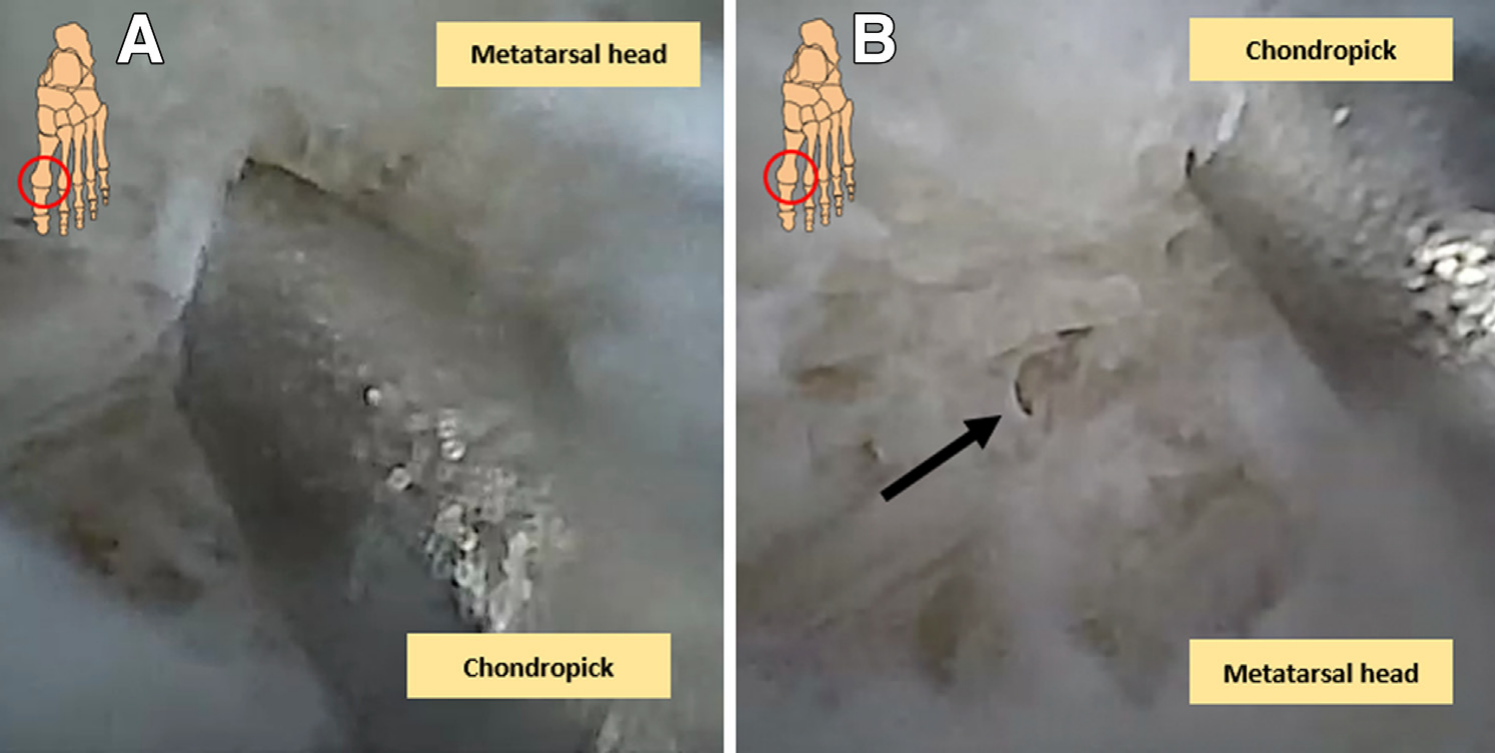
(A and B) Intra-articular chondral picking of the (osteo)chondral lesions of the metatarsal head of the first metatarsophalangeal joint seen with needle arthroscopy. The black arrow indicates a created hole of the (osteo)chondral lesion after microfracturing.

In case of the presence of an (osteo)chondral defect of the metatarsal head, the intervention is started by probing the cartilage borders in order to determine stability and size of the lesion. Any loose chondral flaps are resected, after which debridement of the lesion with a 2-mm diameter shaver blades/burrs or a 3-mm shaver blade takes place just until healthy borders of the surrounding cartilage are reached (Fig 6). Hyperplastic synovium, cicatrized joint capsule, and osteophytes may be resected in a concomitant fashion. Thereafter, under direct visualization, microfracturing of the osteochondral defect through a small chondropick is performed by perforating the subchondral bone plate in order to introduce growth factors and mesenchymal stem cells arising from the bone marrow (Fig 7, A and B). Instruments can be switched between the portals in order to facilitate comprehensive treatment and adequate visualization.

### Closure and Postoperative Protocol

If needed, a final lavage can be performed to clear any debris. The joint is then aspirated to dryness, and all instruments are removed. Sterile wound closure strips or a simple band-aid can be used for wound closure, and a pressure band-aid may be added for comfort and additional hemostasis if required. Postoperatively, because of the minimal invasiveness of the needle arthroscopy procedure, direct passive and active range of motion exercises are encouraged to the patient, and the patient is advised to progressively initiate weight bearing of the foot, as tolerated.

## Discussion

Cartilage and osteochondral lesions of the first metatarsophalangeal joint of the foot are characterized by pain during weight bearing and walking.^6^, ^7^, ^8^ The lesions often require surgical intervention(s). Arthroscopic bone marrow stimulation may be considered the preferred operative intervention for small lesions. Technological advances, patient preferences, and economic considerations combine to foster the development of minimally invasive needle arthroscopic procedures. The present technical note presents a standardized needle arthroscopic approach to the first MTP joint, with the possibility to obtain minimally invasive access to the joint for diagnostic and a variety of concomitant interventional strategies. Recent innovations have increased image quality and made arthroscopic instruments with similarly small diameters.^1^^,^^3^^,^^9^^,^^10^ This substantially improved the diagnostic and interventional capabilities of needle arthroscopy.

Various needle arthroscopic techniques have been performed in multiple joints for diagnostic and therapeutic approaches.^2^, ^3^, ^4^^,^^9^^,^^10^ However, literature on needle arthroscopy of the first MPT joint is lacking.^5^^,^^6^ The use of needle arthroscopy, with its small diameter, leads to less damage to surrounding soft tissue, resulting in improved cosmetic outcomes and reduced wound complications compared to traditional arthroscopy or arthrotomy. As already published in several studies, needle arthroscopy can be performed under local anesthesia.^9^^,^^10^ Consequently, this stepwise approach can also be used to perform MTP-needle arthroscopic procedures under local anesthesia, with the additional use of lidocaine 2.0% injected along the inflow and outflow portal tracts from the skin to the joint capsule and intra-articularly. For example, Kaplan et al.^5^ performed a needle arthroscopic cheilectomy for hallux rigidus in the office setting. However, larger studies are needed to confirm these advantages.

When considering needle arthroscopy for patients with MTP-1 problems, it is important to consider potential pitfalls and disadvantages (Table 2). The introduction of needle arthroscopy can be difficult in small joints. Furthermore, the 0° inclination (rather than the 30° inclination in conventional arthroscopy) can result in a more difficult overview of the joint, which may be unfamiliar to surgeons and needs a small learning curve. Besides, it is possible that the needle arthroscopic procedure is hampered by different reasons (e.g., osteoarthritis of the joint, poor vascular status, soft-tissue compromise, and big osteophytes preventing the introduction of the scope).

**Table 2:** Pearls and Pitfalls

Pearls	Pitfalls
Through an adequate history, physical examination, and a preoperative computed tomography scan of the foot, the needle arthroscopic procedure provides a minimally invasive alternative to conventional arthroscopy for the small MTP-1 joint, enhancing the postoperative rehabilitation and joint stiffness.	Incorrect portal placement, causing iatrogenic (neurovascular) damage or damage to the extensor tendon
Careful placement of the dorsolateral portal under direct arthroscopic visualization	To avoid postoperative stiffness of the MTP-1 joint, prolonged postoperative immobilization should be avoided.
Decreased postoperative pain levels due to minimally invasive procedure	Iatrogenic damage to articular cartilage of the MTP-1 joint from trocar placement

MTP, metatarsophalangeal.

In conclusion, the technique presented here provides a standardized approach for needle arthroscopy of the first MTP joint, which facilitates a safe, uniform, and beneficial adoption of this emergent technique.

## Supplementary Data

ICMJE author disclosure forms

Video 1 Video 1 presents a standardized approach to needle arthroscopy of the first metatarsophalangeal joint using the needle arthroscopy system for osteochondral lesions of the first metatarsophalangeal joint through a step-by-step approach.
